# Breastfeeding duration in mothers who express breast milk: a cohort study

**DOI:** 10.1186/1746-4358-1-28

**Published:** 2006-12-22

**Authors:** Nwet N Win, Colin W Binns, Yun Zhao, Jane A Scott, Wendy H Oddy

**Affiliations:** 1 School of Public Health, Curtin University, Perth, Australia; 2 Division of Developmental Medicine, Human Nutrition Section, University of Glasgow, UK

## Abstract

**Background:**

The expression of breast milk allows a mother to be away intermittently from her infant while continuing to breastfeed. The aim of this study was to investigate the association between expression of breast milk and breastfeeding duration

**Methods:**

A cohort study of 12 months duration. The mothers were recruited from two public maternity hospitals in Perth, Australia between mid-September 2002 and mid-July 2003. While in hospital, participating mothers completed a questionnaire that included questions on how they were feeding their newborn. Telephone interviews conducted at regular periods monitored changes in infant feeding practices, including expression of breast milk. Multivariate Cox regression analysis was used to explore the association between breast milk expression and the duration of any breastfeeding.

**Results:**

A total of 587 mothers, or 55% of those eligible, participated in the study. Of these 93.5% were breastfeeding at discharge from hospital. Mothers who expressed breast milk (at one or more time periods) were less likely to discontinue any breastfeeding before six months (Relative Risk 0.71, 95% CI 0.52, 0.98) than those who had never expressed milk.

**Conclusion:**

This study found that mothers who express breast milk are more likely to breastfeed to six months (any breastfeeding). While further research is required in different cultures to confirm these results, the appropriate use of expressed breast milk may be a means to help mothers to achieve six months of full breastfeeding while giving more lifestyle options.

## Background

Breastfeeding is the preferred way to feed all infants and expressing breast milk allows mothers to be away intermittently from their infants to exercise lifestyle choices while continuing to breastfeed. A recent cohort study from Perth, Western Australia showed that while the breastfeeding initiation rate was 93%, by six months only 46% of mothers were still breastfeeding [1,2].

As in most industrial countries the trend in Australia is for mothers to return to work after having children. The participation rate of women in the Australian work force has increased from 51 to 54% in the decade to year 2000 [3]. The percentage of females in the workforce with children aged 0–4 years increased from 46 to 49% during this period. The most common employment arrangement in families is for both parents to be working and in 2000, 63% of couple families with dependent children had both partners employed [3]. In Australia unpaid maternity leave of 52 weeks following the birth of a child (where the employee had 12 months continuous service with the one employer) was made available to women employed under federal awards in 1979 and under state awards soon afterwards [4]. O'Neill reviewed the availability of paid maternity leave in Australia and he found that 39% of female employees in Australia can take an average of seven weeks' paid maternity leave [5]. Among developed countries, only the US and Australia have not legislated for minimum paid maternity leave across the workforce [5].

Return to work by mothers before their infant is 12 months old is negatively associated with breastfeeding duration amongst Australian women [2]. But this is not the case in some developing countries as a survey in Kenya showed. In Nairobi mother's were able to return to paid employment by expressing milk for a daytime feed [6].

Mothers who want to return to work and to continue breastfeeding usually express breast milk [7-10]. Most recent publications on breast milk expression have included reference to the use of expressed breast milk when mothers return to employment [8,11]. Improved understanding of the mechanism of lactation has lead to improvements in the manufacture of breast pumps and this has made it easier for mothers to express breast milk [12].

If breastfeeding duration is to be maintained or extended at a time where there is a trend towards increasing employment of mothers, breast milk expression has an important role to play. The aim of this paper was to assess the relationship between expression of breast milk and breastfeeding duration.

## Methods

The Perth Infant Feeding Study Mark II (PIFS II) was undertaken in 2002–4 to provide detailed information on breastfeeding in Australia [13]. The study was undertaken in two urban public hospitals in Perth, Australia and because of this the sample is slightly biased towards lower socio-economic groups. In PIFS II 587 mothers who gave birth between mid-September 2002 and mid-July 2003 completed an initial questionnaire while in hospital relating to infant feeding practices and preferences. They were then followed up by telephone interview at approximately 4, 10, 16, 22, 32, 40 and 52 weeks postpartum. When an event of interest occurred, such as a change in the type of breastfeeding or the introduction of solids, the age of the infant was recorded to the nearest week. Over the 12 months of the study, 3976 follow-up interviews were conducted, 85% of the projected number. Further details of the sample and results have been described in the project report and a number of publications [2,13,14].

The project was approved by the research ethics committees of the participating hospitals and by the Human Research Ethics Committee of Curtin University. All mothers were given an information sheet about the study and those willing to participate gave their signed informed consent before being asked to complete the questionnaires. The data were coded and analysed using the SPSS package (SPSS Inc., Chicago, IL, USA). Cox-regression was applied to model the time-to-event data and to determine which variable(s) had an effect on the duration of breastfeeding, for those mothers who were breastfeeding at the time of discharge from hospital.

In this paper breastfeeding is defined as "any breastfeeding" and included infants who were being exclusively, fully or partially breastfed [15].

## Results

The demographic details of the sample are shown in Table 1. In this study 29% of the mothers gave birth by caesarian section. The breastfeeding rate on discharge from hospital was 93%. Comparison with local perinatal statistics suggests that the PIFS II sample was representative of new mothers in Western Australia, with the exception that slightly more mothers smoked in this study [16]. In 2003 in WA the average age of mothers was 29.3 years compared to 28.4 years in this study. In the PIFS II study, 37% of the mothers were primiparous compared to 41% for the whole state. Caesarean section births were 30.9% for WA compared to 29.3% in this study. In WA 18.9% of mothers smoked during pregnancy compared to 26% in this study [16]. The number and proportion of mothers who continued to breastfeed (any and full breastfeeding) during the study are shown in Table 2.

**Table 1:** Demographic characteristics of the participants in the Perth Infant Feeding Study II

	**Total sample (n = 587)**	**% of total sample**	**% who expressed at 4 weeks**
Maternal age (years)			
<20	32	5.5	62
20–29	292	20.8	57
30+	262	30.4	47
Maternal education			
Did not complete high school	211	35.9	51
Completed high school or equivalent*	307	52.3	52
Bachelor degree or higher	69	11.8	55
Parity			
Primiparous	216	36.8	68
Multiparous	371	63.2	44
Birthweight (g)			
<2500	13	2.2	82
>2500	566	97.8	52

* Includes those with a trade or technical certificate/diploma.

**Table 2:** Breastfeeding rates in the Perth Infant Feeding Study II

**Age (weeks)**	**Total sample**	**Any breastfeeding**		**Full breastfeeding**	
	n*	**%**	**n**	**%**	**n**
4	528	79.8	421	32.5	172
10	507	66.6	338	22.2	113
16	486	63.0	306	11.4	55
22	483	51.3	248	4.3	21
32	465	39.3	183		
40	465	33.3	155		
52	455	22.1	101		

* sample size, number of mothers remaining in the study

At each follow-up interview the mothers were asked if they had expressed breast milk since the last interview, details of the methods used and any difficulties encountered. Using a manual pump was the method most commonly chosen by mothers, ranging from 64% at week 4 to 67% at week 22. About 20% of mothers chose to use electric pumps and the remainder used manual expression. The percentage of breastfeeding mothers who had expressed breast milk by each time period covered by the Perth Infant Feeding Study II are shown in Table 3.

**Table 3:** Proportion of breastfeeding mothers who had expressed breast milk by each follow-up interview

**Follow-up interview (Weeks postpartum)**	**Any breastfeeding**	**Ever expressed breast milk**	
	**n**	**%**	**n**
4	421	76.4	322
10	338	61.4	208
16	306	81.5	249
22	248	83.8	208
32	183	67.1	123
40	155	71.9	111
52	101	66.7	67

To determine variables associated with the duration of any breastfeeding, a Cox regression analysis was carried out on those mothers who were breastfeeding at the time of discharge from hospital. The variables included are listed beneath Table 4 and all variables in the final model were variables for which when excluded the change in deviance compared with the corresponding chi-square test statistic on the relevant degrees of freedom was significant at the 5% level. The forward selection procedure was applied in the analysis.

**Table 4:** Association of expression of breast-milk with the risk of discontinuing any breastfeeding *before 6 months *after adjustment for potential confounders* in the Perth Infant Feeding Study II

**Expressed before six months**	**Crude RR**	**(95% CI)**	**Adjusted RR**	**(95% CI)**
Never expressed	1 (reference)		1 (reference)	
Expressed	0.58	(0.45, 0.74)	0.71	(0.52, 0.98)

-2 log likelihood (deviance) 2057.423, df 15

* The variables controlled for the model were infant sex, infant birth-weight, infant admitted in a special care nursery, maternal marital status, demand feeding, parity, attendance at antenatal classes, maternal age, maternal IOWA score, mothers' occupations, age of infant when mother returned to work, maternal years of education, maternal country of birth, how many times mother being sick in one year, maternal grandmother's feeding preference, father's feeding preference, age by which dummy was introduced, time at which infant feeding method decided, how many times mother having breastfeeding problems in one year, and total number of weeks for expression in one year.

**Figure 1:**
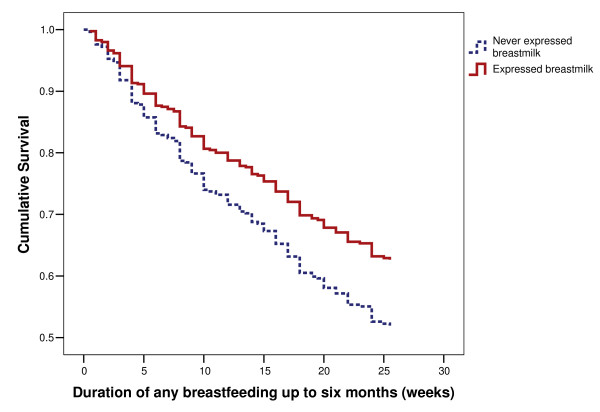
Survival curve of duration of any breastfeeding by mothers who expressed breast milk.

In the model the significant variables that promoted 'any breastfeeding to six months were the age of the mother, not returning to work before six months, heavier birth weight, the use of a pacifier before four weeks, a higher IOWA score and a father who supported breastfeeding [2]. Mothers who had more breastfeeding difficulties were likely to cease earlier. When these factors were adjusted for in the final model (Table 4), expression was found to be associated with 'any breastfeeding' at six months. Specifically mothers who expressed breast milk were less likely to stop any breast feeding before six months compared to those never expressed breast milk. The corresponding survival curve is shown in Figure 1. A log-rank test (χ^2 ^score = 21.081, p value = 0.00004) was performed to identify the difference between the duration of any breastfeeding up to six months for groups of mothers who expressed or not expressed milk.

## Discussion

More than three quarters of mothers practiced breast milk expression in the first month after birth, a rate that is similar to other comparable countries [10]. Expression allows a mother some independence from directly feeding her infant and may allow her to return to work or undertake some social activities while continuing to breastfeed. Biagioli has reviewed workplace issues for mothers wishing to return to paid employment and has described the importance of workplace environments that facilitate breast milk expression [9]. In the USA, mothers employed by companies that sponsored lactation programs that allowed for expression of breast milk had high rates of continued breastfeeding [7].

Some mothers chose to hand express but most mothers used breast pumps to facilitate breast milk expression. In Australia there has been a trend for an increasing number of mothers to express breast milk, but the overall proportion using hand pumps has remained fairly constant at around 65% [17]. There have been many advances in breast pump technology and usage in recent years [18]. Health professionals responsible for education programs need both to be skilled in the teaching of hand expression and knowledgeable of the breast pump technologies available [19]. The usage of pumps requires continuing support, particularly in the first few weeks of use [20].

One advantage of this study was its prospective design, which eliminates problems with mothers' recall. However there is a limitation in the sample selection that needs to be considered when interpreting the results of this study. The sample was slightly biased towards lower socio-economic groups where mothers' rates of return to work are likely to be lower. We cannot exclude the possibility that expression may be a surrogate for another unknown factor that has a positive relationship with breastfeeding, although we have attempted to adjust for known confounders.

## Conclusion

This study found that mothers who express breast milk are more likely to breastfeed to six months ('any' breastfeeding). While further research is required in different cultures to confirm these results, the appropriate use of expressed breast milk may be a means to help mothers to achieve six months of 'full' breastfeeding while giving more lifestyle options. Many antenatal programs now include information on breast milk expression and this could be expanded to ensure that all parents are given information on the appropriate and safe use of expression and the storage of breast milk.

## Competing interests

The author(s) declare that they have no competing interests.

## Authors' contributions

NW: data collection, data analysis and paper writing.

CWB: principal investigator, project design, data analysis, paper writing and revision.

YZ: data analysis.

JS: project design, data analysis and paper writing.

WO: project design, data analysis and paper writing.
